# The use of Veno-arterial Extracorporeal Membrane Oxygenation (VA-ECMO) for Acute High Risk Pulmonary Embolism: A Systematic Review

**DOI:** 10.2174/011573403X339627241224085451

**Published:** 2025-02-10

**Authors:** Rohit Munagala, Humail Patel, Pranav Sathe, Avneet Singh, Mangala Narasimhan

**Affiliations:** 1 Department of Internal Medicine, North Shore University Hospital, Northwell, New Hyde Park, NY, 11030, United States of America;; 2 Long Island Jewish Medical Center, Northwell Cardiovascular Institute, Northwell, New Hyde Park, NY, 11040, United States of America;; 3 Department of Pulmonary and Critical Care Medicine, Long Island Jewish Medical Center, North Shore University Hospital, Northwell, New Hyde Park, NY, 11030, United States of America

**Keywords:** Pulmonary embolism, veno-arterial extracorporeal membrane oxygenation, bridge therapy, solo therapy, hypoxia, thrombolysis

## Abstract

**Introduction:**

Pulmonary embolism (PE) associated with hemodynamic compromise, termed high-risk or massive acute PE (MAPE), is associated with increased morbidity and mortality. Despite advancements in procedural techniques and an increase in the availability of advanced therapies, the outcomes associated with high-risk PE remain poor. Here, we review the literature surrounding the use of Veno-arterial Extracorporeal Membrane Oxygenation (VA-ECMO), primarily as a bridging therapy, in patients presenting with high-risk pulmonary embolism.

**Methods:**

We conducted a systematic review and meta-analysis utilizing PubMed/MEDLINE from database inception until March 2024. The terms “high-risk PE”, “massive PE” and “MAPE” were paired with “VA-ECMO”, “bridge therapy” and “solo therapy” along with related terms to find and analyze relevant studies. The primary outcome assessed was in-hospital mortality.

**Results:**

Most comparative studies involved assessing VA-ECMO's utility as solo therapy *vs* as a bridge to advanced therapy. Out of the data involving VA-ECMO as solo therapy, most showed definite survival benefit in subset of populations with VA-ECMO's role being varied by age and cardiac arrest presence. A portion of studies were notable for finding no difference in outcomes; however no major retrospective determined negative effect of VA-ECMO. In head-to-head studies as a bridge, studies from multiple centers highlighted VA-ECMO's role in stabilizing and improving survival in massive PE prior to systemic or catheter directed thrombolysis. Follow-up studies were limited, however one retrospective showed 30-day mortality of 31% and the 1-year mortality of 54% post PERT call. Follow-up echocardiograms performed on survivors between 30-365 days from Pulmonary Embolism Response Team (PERT) activation interestingly all had normal Right Ventricular (RV) size and function with mild to no tricuspid regurgitation.

**Conclusion:**

Most major literature supports the use of VA-ECMO as either solo therapy or a bridge to advanced therapy in MAPE with additional shock or cardiac arrest. However, further studies are needed to develop society guidelines for regular initiation in cases of MAPE.

## 1 INTRODUCTION

Pulmonary embolism (PE) associated with hemodynamic compromise, termed high-risk or massive acute PE (MAPE), is associated with increased morbidity and mortality [1]. Approximately 5% of patients with PE are categorized as high risk [2, 3]. Clinical manifestation of massive PE can evolve rapidly with progression of right ventricular (RV) failure, hypoxia, shock and cardiac arrest. 90-day mortality of patients with high-risk PE has been demonstrated to be as high as 50% in some studies [4, 5].

The degree of the patient’s shock state due to high-risk PE is dependent on the severity of the development of acute right ventricular (RV) failure [6]. Echocardiography is utilized to evaluate right ventricular failure, which is indicated by right ventricular dilation (Right Ventricle/Left Ventricle ratio > 1:1), septal bowing, and the McConnell sign (characterized by hypokinesis of the basal and mid right ventricular free wall while maintaining apical contractility) [7]. Physiologically, obstructed pulmonary vasculature leads to a profound increase in right ventricular wall stress, myocardial ischemia, and decreased right ventricular function, resulting in obstructive shock. Other anatomic complications of this mechanical obstruction include reduced left ventricular preload and septal bowing from a dilated right ventricle that compromises left ventricular function and cardiac output [8]. Concomitantly, an increase in alveolar dead space and heightened ventilation-perfusion mismatch because of diminished pulmonary arterial blood supply may exacerbate and trigger impending cardiopulmonary compromise [9].

**Fig. (1):**
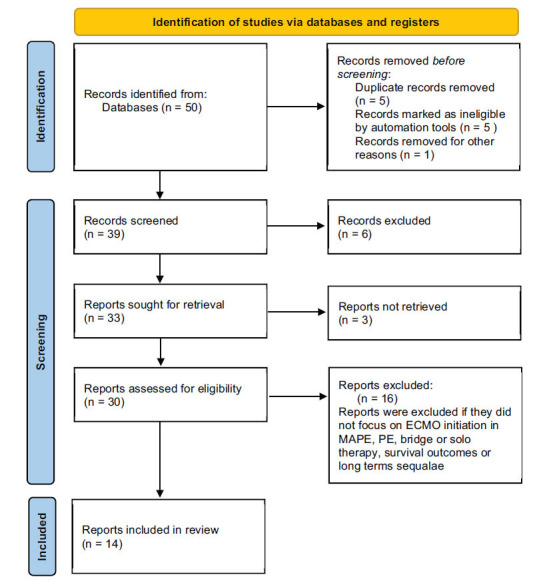
The above figure demonstrates identification of studies *via* databases and registers.

Due to the potential for rapid decompensation, prompt initiation of a multimodality treatment regimen, consisting of cardiopulmonary support, anticoagulation, and reperfusion, is critical to achieving a favorable outcome in MAPE (Fig. **1**) [10]. Anticoagulation, primarily with unfractionated heparin, is standard therapy, and is essential to both hinder propagation of the known embolism and to prevent the development of additional emboli while reperfusion therapy is initiated [11, 12]. Rapid reperfusion therapy allows for the restoration of pulmonary blood flow with a resulting decrease in pulmonary pressures and resistance, to ultimately allow for recovery of right ventricular function [13]. This rapid improvement in pulmonary obstruction, *via* rapid reperfusion compared to unfractionated heparin alone, also leads to a faster reduction in RV dilatation, indicating better response [14, 15]. Intravenous thrombolytic therapy, most often *via* tenecteplase or alteplase, is often considered first-line therapy with intent to achieve immediate reduction of thrombotic burden. This therapy has been shown to reduce total mortality, PE recurrence, and PE-related mortality [16]. However, in patients in whom systemic thrombolysis may be contraindicated or patients who have failed systemic thrombolysis, catheter-guided thrombectomy has been shown to be an effective therapeutic option for MAPE [17-20]. Surgical pulmonary embolectomy may also be offered in specific scenarios, such as in patients with extensive proximal thrombus burden with hemodynamic compromise, patients who experience clot-in-transit, patients who have contraindications to other available therapies, or in those patients where offered modalities fail [21, 22]. Recent research and reports have indicated a favorable outcome for patients undergoing surgical pulmonary embolectomy in MAPE [23, 24].

Despite reperfusion therapies as described above, patients may present with hemodynamic collapse necessitating veno-arterial extracorporeal membrane oxygenation (VA-ECMO) support to provide complete cardiopulmonary support in critical situations. VA-ECMO’s instant cardiopulmonary support facilitates hemodynamic stabilization and systemic perfusion, which is essential for survival [25]. The first reported case of high-risk PE managed with cardiopulmonary bypass, in conjunction with surgical removal of the clot, was described in 1961 [26]. Since then, with the advent of modern ECMO and increased availability, it has become a more viable option in dire situations. VA-ECMO is primarily used as a bridging therapy to provide cardiopulmonary support while reperfusion therapy is pursued [27]. The utility of VA-ECMO in patients with hemodynamic compromise due to high-risk PE centers around a scenario in which RV failure and resulting catastrophic cardiogenic shock have already occurred and thus, advanced cardiopulmonary support is indicated [28]. The use of VA-ECMO as a stand-alone therapy by allowing time for physiologic thrombolysis to occur while administering anticoagulation has also been explored, though data is sparser in this sphere [29]. The available data does suggest VA-ECMO initiation with reperfusion strategies reduces in-hospital mortality compared to VA-ECMO stand-alone therapy [30]. Overall, the use of VA-ECMO stand-alone therapy for MAPE is controversial [29, 31]. More recent societal guidelines, including those of the European Society of Cardiology and American Heart Association, are beginning to list VA-ECMO as a possible consideration for patients with refractory circulatory collapse while reperfusion therapy *via* surgical embolectomy or catheter-directed treatment is pursued [32, 33].

Though VA-ECMO is becoming more popular in scenarios of hemodynamic instability because of high-risk PE, the question arises whether veno-venous extracorporeal membrane oxygenation (VV-ECMO) has any utility in the treatment of these patients. While circulatory compromise is primarily driven by right ventricular failure, profound respiratory failure, and refractory hypoxia due to alveolar dead space and ventilation-perfusion mismatch may rarely be encountered as well. In situations where mechanical ventilation is not sufficient to correct this hypoxia, VV-ECMO has been utilized, often after reperfusion has already been achieved [34]. It is notably rare, however, to have refractory respiratory failure in the absence of circulatory failure and thus, VA-ECMO remains to be the ECMO mode of choice [35].

It is exceedingly recognized that pulmonary embolism, especially intermediate and high-risk PE patients have poor outcomes. There has been a rapid evolution of therapeutic interventions with a favorable impact on patient outcomes. The need for multidisciplinary approach in tailoring management decision for this heterogenous, complex and critical patient group has led to adoption of pulmonary embolism response teams (PERTs) across healthcare systems, The purpose of PERTs is to allow for the immediate deployment of several specialists to discuss treatment options and ultimately develop a management plan for patients presenting with pulmonary embolism [36, 37]. The structure of the PERT varies by institution but often contains specialists from several fields including critical care medicine, interventional and noninvasive cardiology, cardiac surgery, pulmonary medicine, vascular surgery, and interventional radiology. Prompt recruitment of a multispecialty team may facilitate the decision to pursue more appropriate therapies such as catheter-directed treatment, surgical embolectomy, or the decision to place a patient on VA-ECMO. Studies have demonstrated that institutions with a dedicated PERT will frequently offer and pursue advanced therapies, albeit being unclear if the PERT response algorithm improves clinical outcomes [38].

Despite advancements in procedural techniques and an increase in the availability of advanced therapies, the outcomes associated with high-risk PE remain poor [39]. The utility and efficacy of VA-ECMO in the setting of hemodynamic compromise have been demonstrated primarily in case series and reports, but large-scale data and randomized controlled trials are lacking. Herein we present an updated review of the literature surrounding the use of VA-ECMO, primarily as a bridging therapy, in patients presenting with high-risk pulmonary embolism, a topic that remains without standard of care or consensus guidelines. Of note many of the listed studies define high-risk PE as massive acute PE (MAPE) as per prior guidelines; for the sake of simplicity, we will be utilizing both these terms interchangeably.

## 2 METHODOLOGY

### 2.1 Literature Search

Literature searches utilizing PubMed/MEDLINE from database inception until March 2024 were conducted. The terms “high-risk PE”, “massive PE” and “MAPE” were paired with “VA-ECMO”, “bridge therapy” and “solo therapy” along with “ RVAD” and “MCS” related terms to find and analyze relevant studies. Relevant filters were applied to ensure fully published full-text, English-language studies with human subjects.

### 2.2 Study Selection

Two independent reviewers (H.P., R.M.) assessed each paper for eligibility according to criteria for inclusion and exclusion. Inclusion criteria were broad and included any retrospective studies or meta-analysis discussing MAPE or high-risk PE along with treatment, specifically MCS that included VA-ECMO and RVAD. Search criteria were filtered to full-text, English-language studies with human subjects. Exclusion criteria were based on if studies did not mention PE, VA-ECMO and/or RVAD. Both reviewers independently examined each study slated to be included in the review to exclude any bias. No automation tools were used in the analysis and summarization of any involved study.

### 2.3 Data Selection

Outcomes of interest from each study included complications of PE, method of treatment chosen for PE, from reperfusion therapies of choice to thrombolytics, VA-ECMO or RVAD, whether VA-ECMO was chosen as solo or bridge therapy, overall mortality rate of each treatment option and complications from initiating VA-ECMO. Each study's results were collected by two authors (H.P, R.M.) and analysis and conclusions were cross-referenced for each study by both authors. The Mixed Method Appraisal Tool (MMAT) was utilized to assess risk of bias for each study utilized and analyzed.

## 3 RESULTS

### 3.1 VA-ECMO Solo Therapy

An observational study assessing 219 patients from the National Inpatient Sample (NIS) database from 2005-2013 found among patients with high-risk PE, there was lower in-hospital mortality with VA-ECMO use (OR 0.34 [95% CI=0.25-0.^45^], *p<* 0.001) via multivariate regression analysis. Additionally, models for patients with the following qualities were statistically significant as predictors of in-hospital mortality: older age, female sex, heart failure, chronic pulmonary disease, and obesity. Among those with high-risk PE on VA-ECMO 89% were intubated, 66% developed renal failure, and 27% needed a blood transfusion [40].

The use of mechanical support devices (VA-ECMO, RVAD) in patients with massive acute pulmonary embolism (MAPE) who were poor candidates or failed established advanced therapy (systemic/catheter-directed thrombolysis, surgical embolectomy) was investigated in a 2020 review at Yale University. After a review of 16 studies, 164 cases of VA-ECMO were identified with the primary outcome time to hospital discharge. 122 (74%) of the patients on VA-ECMO were able to be weaned from support, with 120 (73%) surviving until hospital discharge. The study concluded there was a benefit in using any sort of MCS for patients with massive PE who are poor candidates for conventional interventions or with refractory PE [41]. A meta-analysis conducted at the University of Virginia in 2021 directly compared mortality in MAPE between those with and without VA-ECMO. There was no differentiation of VA-ECMO candidates between those utilizing it as solo therapy *vs* those being bridged to advanced therapy. Out of 791 MAPE patients over 11 studies from 1992-2002, 270 were cannulated to VA-ECMO and 521 were not. It was observed that mortality was not significantly different between both populations (OR 1.24 [95% CI 0.63–2.^44^], *p*=0.54), though further subgroup analysis was needed to address population heterogeneity [42]. This is supported in a retrospective cohort study of 40 patients with high-risk PE at Kaohsiung Chang Gung Memorial Hospital, which found no significant difference in major complications and in-hospital mortality between VA-ECMO and non-VA-ECMO treated groups. In subgroup analysis, earlier VA-ECMO treatment was associated with a reduced risk of cardiac arrest (*p*=0.023), lower in-hospital mortality (*p*=0.036), and higher overall survival (*p*=0.033) when compared to patients treated without VA-ECMO. These results were specific to patients with hemodynamic instability but without cardiac arrest. Those patients needing CPR had higher in-hospital mortality without survival benefit on VA-ECMO. As such, the study concluded early VA-ECMO intervention among hemodynamically unstable patients without cardiac arrest should be considered [43].

In contrast, a 2021 meta-analysis at the University of Amsterdam concluded only a limited utility of VA-ECMO in acute PE. The study collected the use of VA-ECMO in acute PE with obstructive or cardiogenic shock. Results were notably divided by age less than or greater than 60 years, cardiac arrest pre or post VA-ECMO cannulation, and whether VA-ECMO was used alone or as a bridge to systemic thrombolysis or surgical embolectomy. Twenty nine observational studies were grouped with a total 1138 patients treated with VA-ECMO. 809 patients were considered control with no use of VA-ECMO. Analysis of short-term survival (hospital stay or 30 days) showed no difference between VA-ECMO and control groups (RR 0.91 [95% CL 0.71-1.^16^]). Among VA-ECMO subgroups, lower survival was seen among patients > 60 years (RR 0.72 [95% CI 0.52-0.99]) and those with pre-ECMO cardiac arrest (RR 0.88 [95% CI 0.77-1.0^1^]), while ECMO bridged to surgical embolectomy had higher survival (RR 1.96 [95% CI 1.39-2.76]). Among those with cardiac arrest treated with VA-ECMO, short-term survival was around 34%, notably higher than the survival rate of those with cardiac arrest without ECMO (survival 8.5–18.3% as reported in the literature). Despite the lack of evidence supporting the use of VA-ECMO in overall acute PE groups, the study postulated that there is a possible benefit of VA-ECMO in those subgroups with cardiac arrest from acute PE requiring immediate support [44].

These results were similar to conclusions drawn from a 2020 systematic review at Temple University, which included 301 patients over 77 articles. It was noted that VA-ECMO in massive PE-related cardiac arrest had a 61% survival. Major takeaways included survival rates being similar regardless of whether patients received systemic thrombolysis before cannulation *vs* not receiving systemic thrombolysis at all (67% *vs* 61%, *p*=0.48). There was increased risk of mortality in patients > 65 years (OR=3.08) and if cannulation occurred during cardiopulmonary resuscitation (OR=5.67) [45].

### 3.2 VA-ECMO Solo *versus* Bridge Therapy

A retrospective analysis assessed outcomes of VA-ECMO use in PE-related cardiogenic shock/arrest between 2005-2018 in Germany. Mortality was analyzed with the use of VA-ECMO alone (n=588), VA-ECMO and thrombolysis (n=165), and VA-ECMO and embolectomy (*n*=385). When compared to a control of thrombolysis alone (OR 1.04 [95% CI 0.99-1.0^1^], *p*=0.116), multivariable logistic regression analysis indicated lower risk for in-hospital death in VA-ECMO alone (OR 0.68 [95% CI 0.57-0.82], *p*<0.001), VA-ECMO with thrombolysis (OR 0.60 [95% CI 0.43-0.85], *p*=0.003), and VA-ECMO with embolectomy (OR 0.50 [95% CI 0.41-0.61], *p*<0.001). The study concluded that VA-ECMO alone or in tandem with advanced therapy has benefits for PE with cardiac arrest [46]. Similarly, a 2019 case series at Asan Medical Center monitored the results of VA-ECMO initiation in 16 patients with cardiovascular and respiratory collapse. Patients either received VA-ECMO alone (n=3) or were bridged to systemic thrombolysis (n=4), surgical embolectomy (n=6), or both (n=3). Overall, 30-day mortality was 43.8% with subgroup mortalities of 33.3% in VA-ECMO alone (n=3), 50.0% in VA-ECMO bridged to thrombolysis (n=4), and 44.4% in VA-ECMO bridged to embolectomy (n=9). Cardiac arrest was seen before cannulation in 12/16 cases. Given that cardiac arrest is an exacerbating risk factor for death from PE (mortality ranging from 52.0-65.0% per literature), the overall mortality observed in this study may represent some utility of VA-ECMO as rescue therapy for those with cardiac arrest or refractory shock [47]. At the University of New Mexico, 17 patients with massive PE were placed on VA-ECMO for hemodynamic stabilization between 2017 and 2019. All the patients were in shock, differentiated into cardiogenic *vs* obstructive. Overall survival was noted in 13 of the 17 patients, 12 of whom were eventually discharged without noted neurologic damage. Of the survivors, 10 of 13 required anticoagulation in conjunction with VA-ECMO; the remaining three required thrombectomy in addition to anticoagulation due to right heart function. The results strongly supported use of VA-ECMO, regardless of adjunctive reperfusion therapy, as part of efforts to stabilize patients with MAPE [48].

A direct comparison of VA-ECMO monotherapy *vs* VA-ECMO and systemic/catheter-directed thrombolysis was analyzed in a 2021 retrospective at Geneva University Hospital. Over 10 years, 36 patients with MAPE and obstructive and/or refractory cardiogenic shock were cannulated. Systemic and/or catheter-directed thrombolysis was conducted in 17 of these patients (systemic in 16 and catheter-directed in 5 patients). Surgical embolectomy was not offered. Overall survival was 64%, noteworthy when compared to the mortality in MAPE which was up to 50%. Within 24 hours, most patients had either hemodynamic or respiratory improvement, and 30-day survival was noted to be improved (OR 15.58 [95% CI 2.65-91.57]). When VA-ECMO was utilized after the failure of thrombolysis therapy, there were noted bleeding complications (17 (100%) *vs*. 1 (5.3%) patient, *p* < 0.001) and a worse 30-day mortality (OR 0.11 [95% CI 0.022-0.5^20^], *p*=0.006). There was an increased risk of death in subgroups with thrombolysis failure, VA-ECMO cannulation during CPR (*vs* not), and the presence of cardiac arrest before VA-ECMO initiation (*vs* not). The most interesting finding involved 30-day mortality being significantly higher in patients receiving VA-ECMO and thrombolysis compared to VA-ECMO alone (64.7%, n=11/17 *vs* 10.5%, n=2/19, *p*=0.001). Overall the study supported that short-term mechanical circulatory support allows for stabilization in critically ill patients with MAPE [49].

Survival benefit with a VA-ECMO bridge to surgical embolectomy was observed in a multicenter study conducted in France, assessing 180 patients from 2014-2015 with high risk PE. Out of the patients, 128 were treated without VA-ECMO compared to 52 undergoing cannulation. Overall 30-day mortality was 48.3%. Upon subgroup analysis, mortality was 43% in those treated without VA-ECMO *vs* 61.5% in those with VA-ECMO (*p*=0.008). VA-ECMO mortality was further divided based on concomitant therapy. Thirty-day mortality was 77.7% (n=14/18) for VA-ECMO alone, 76.5% (n=13/17) for VA-ECMO and fibrinolysis, and 29.4% (n=5/17) for VA-ECMO and surgical embolectomy (*p*=0.004). Among patients with VA-ECMO, 38.5% had a major bleeding event in-hospital. The study concluded that VA-ECMO alone or with failed fibrinolysis has a statistically worse prognosis compared to VA-ECMO with surgical embolectomy, which should be considered as potential therapy [29].

### 3.3 Complications and Long Terms Effects

A retrospective study of MAPE treated with VA-ECMO from 2015-2020 assessed the safety of cannulation after recent systemic thrombolysis. Out of the 83 patients with MAPE on VA-ECMO, 18 received adjunctive systemic thrombolytics with no significant difference in survival compared to those without thrombolysis (88.9% *vs* 84.6%; *p* = 0.94). There was no significant difference in time on mechanical ventilation, need for renal replacement therapy, or length of stay between the groups. However, there was a higher incidence of major bleeding events in patients with recent systemic thrombolytics (61.1% *vs* 26.2%; *p* = 0.01). The study concluded that VA-ECMO in close proximity to anticoagulation can still be considered reasonable despite this risk and that systemic thrombolysis should not be considered a contraindication to cannulation for VA-ECMO [50].

A 2017 retrospective at the Hôpital de la Pitié–Salpêtrière serves as the largest modern-day analysis on long-term survival for patients with MAPE treated with VA-ECMO. Out of 17 cannulated patients from June 2006-June 2015, 47% (n =8) survived past 90 days of ICU discharge. After a median follow-up of 19 months post-ICU discharge, while general and mental health were normal per survey, all patients interviewed still had physical debilitations [31]. At Massachusetts General Hospital, a case series of 13 patients with cardiac arrest and MAPE treated with VA-ECMO from 2012-2017 assessed 30-day and 1-year mortality. All patients showed right ventricular (RV) dilation and hypokinesis on echo before cannulation. Adjunctive therapies were as follows: 62% received systemic thrombolysis, 23% underwent catheter-directed thrombolysis, 8% received both, and 31% underwent surgical embolectomy. Among these patients, 15% had both systemic tPA and surgical embolectomy. Mortality data was notable for 54% dying within a year of PE, four of whom (57%) died within 30 days. Major bleeding within 30 days of PE was seen in 54% of patients. Nine total patients survived the first 30 days. Seven of these patients had follow up echo within 30 days; 86% had normal RV size and function, with 14% having a mildly dilated RV with hypokinesis. All had normal LV function. Five of the surviving nine also had follow-up echocardiograms performed between 30-365 days from PERT activation, with all having normal RV size and function with mild to no tricuspid regurgitation. Overall, the 30-day mortality was 31% and the 1-year mortality was 54%. While the 30-day mortality remains high, it is to be noted that this study had a lower mortality than other studies. Furthermore, in a follow-up study of patients surviving till hospital discharge, nearly all cases had normal RV parameters on echo. Given that two-thirds of patients survived to the 30-day end point, the study concluded that PERT teams have the ability to expedite the care of unstable people with PE and mobilize appropriate resources for their care, including the use of VA-ECMO. The use of VA-ECMO was concluded to be a suitable solo therapy or bridge to advanced therapy [51].

32 patients with massive PE placed on VA-ECMO were analyzed at the University of Kentucky. 21 (66%) survived till decannulation, with 17 (53%) surviving till discharge. Upon comparison of survivors and non-survivors, there were no differences noted in age, gender, right ventricle-to-left ventricular ratios, or peak troponin-T, though non-survivors did tend to have a malignancy history. Interestingly all patients with adjunctive systemic thrombolysis (n=5) died, while 11/15 patients who received catheter-directed thrombolysis survived. It was noted that cardiac arrest before cannulation had worse outcomes. Additionally, lactate levels < 6 mmol/L were noted to have 82.4% sensitivity and 84.6% specificity for predicting survival to discharge. This study concluded that the use of VA-ECMO should be reserved for those with adequate baseline cardiac function [52].

## 4 DISCUSSION

The management of high-risk pulmonary embolism involves multiple therapeutic approaches simultaneously - namely anticoagulation, reperfusion, and cardiopulmonary support. Given the variabilities in presentation, there is no consensus on how to best manage a patient in hemodynamic collapse as a result of high-risk PE. The use of VA-ECMO as a means of providing cardiopulmonary support in high-risk PE has become an increasingly popular option in scenarios of refractory shock. Given the sparsity of robust data, such as randomized controlled trials or even large-scale observational studies, the true degree of benefit concurred with VA-ECMO is unclear. However, several studies, as outlined above, offer insight into the spectrum of utility of VA-ECMO in these situations and may pave the way for further research, and ultimately institutional guidelines to standardize treatment of this clinical challenge. At present, out of the major institutions offering guidance on the management of high-risk pulmonary embolism, only the European Society of Cardiology recognizes VA-ECMO as a potential consideration, whereas others, such as the American Heart Association do not yet include ECMO as a strong option in the management of high-risk PE due to the scarcity of data [53]. 

VA-ECMO is most often used as a bridging therapy to provide extensive cardiopulmonary support while other, more definitive, therapies are pursued. Though the data, as delineated above, appears to be somewhat inconclusive in this regard, it generally points to a beneficial role of ECMO in these populations. Observational studies at several institutions revealed reduced in-hospital mortality, with or without advanced reperfusion therapies in several cohorts of patients [40, 44, 45]. One conflicting factor was the presence or absence of cardiac arrest prior to cannulation, which was associated with worse or favorable outcomes depending on the population [29, 44, 45]. This may be due to small sample sizes and the already morbid prognosis and high mortality in this subset of the population with hemodynamic instability. In certain instances when a patient is not a candidate for any therapeutic interventions, VA-ECMO may be used as stand-alone therapy to effectively buy time for intrinsic thrombolysis to occur. This situation is far less common and the current data surrounding its outcomes appears to be more inconsistent. Certain studies did, however, suggest that there may be benefit, including lower in-hospital mortality, to using VA-ECMO in patients who are poor candidates for reperfusion therapies [41, 42]. Other studies demonstrated potential benefit only in subgroup analyses, such as those who had not suffered from cardiac arrest [43]. This inconsistency in results may seem more puzzling, but it is to be expected given the critical nature of the patients who are candidates for ECMO to begin with.

Based on conducted reviews, there are no major established exclusion criteria for the use of VA-ECMO in high risk PE separate from the general guidelines. ECMO was utilized as a method to augment cardiac output in settings where there was severe right ventricular dysfunction secondary to the embolic physiology. General exclusion would be unwitnessed cardiac arrest, extended down time without reperfusion, pathologic anatomy (aortic dissection, regurgitation), brain death, and poor functional prognosis based on the patient's past medical history [46, 51-54].

Specific criteria for what constitutes major/minor bleeding differed per case review. Most case studies documented major bleeding as that which resulted in hypotension, required resuscitative transfusion, and/or anatomical bleed (brain bleed, melena, dermatomal extravasation, *etc*.). Minor bleeding was defined as bleeding around the cannulation sites and notable easy bruising. Of note, PTT goals for systemic thrombolysis were not documented in the studies, and could be considered an area of further standardization. While PTT goals in the United States should be based on the recommendation per the American Heart Association, there is to be expected differences in goal PTT values as per individual hospital policy.

It is clear that the use of VA-ECMO has become more popular with its increasing availability and its utility as a rescue therapy in critical situations of hemodynamic compromise. To this date, there are no randomized controlled trials to clearly demonstrate the benefit of ECMO in this setting. Despite observational and single institutional studies showing potential for benefit, more studies are certainly needed to clearly establish the role of ECMO in the management of patients with high-risk PE. 

## CONCLUSION

Most major literature supports the use of VA-ECMO as either solo therapy or a bridge to advanced therapy in MAPE with additional shock or cardiac arrest. It is noteworthy that several of the studies conducted suffer from being single-center studies. There is a degree of selection bias for patients chosen to be cannulated as those requiring VA-ECMO are more likely to be hemodynamically unstable compared to control groups, thus leading to higher mortality at baseline.

Major subgroup analysis is required to find the optimal candidates to cannulate to optimize mortality. For example, it would be helpful to have studies comparing outcomes of cardiac arrest with or without return of spontaneous circulation prior to VA-ECMO cannulation. Additionally, comparing the timing of reperfusion therapies in conjunction to VA-ECMO initiation would provide valuable information. VA-ECMO treatment prior/post advanced therapy can have different outcomes and should be discovered. Furthermore, areas of study will need to assess the development of long-term complications from VA-ECMO, not limited to pulmonary hypertension, CTEPH, and RV remodeling as seen on echo. Additionally, with the advent of the Right Ventricular Assist Device (RVAD), it is reasonable to compare outcomes between VA-ECMO *vs* RVAD devices.

While VA-ECMO has definite potential, further studies are unequivocally needed before developing society guidelines for regular initiation in cases of MAPE.

## Data Availability

The authors confirm that the data supporting this study are available within the article.

## LIST OF ABBREVIATIONS

CTEPH: Chronic Thromboembolic Pulmonary Hypertension
ECMO: Extracorporeal Membrane Oxygenation
MAPE: Massive Acute Pulmonary Embolism
MCS: Mechanical Circulatory Support
PE: Pulmonary Embolism
PERT: Pulmonary Embolism Response Team
RV: Right Ventricle
RVAD: Right Ventricular Assist Device
VA-ECMO: Veno-arterial Extracorporeal Membrane Oxygenation

## STANDARDS OF REPORTING

PRISMA guidelines and methodology were followed.

## SUPPLEMENTARY MATERIAL

PRISMA checklist as a supplementary material is available on the publisher's website along with the published article.

## References

[1] Quezada C.A., Bikdeli B., Barrios D., Barbero E., Chiluiza D., Muriel A., Casazza F., Monreal M., Yusen R.D., Jiménez D. (2019). Meta-analysis of prevalence and short-term prognosis of hemodynamically unstable patients with symptomatic acute pulmonary embolism.. Am. J. Cardiol..

[2] Kahn S.R., de Wit K. (2022). Pulmonary embolism.. N. Engl. J. Med..

[3] de Wit K., D’Arsigny C.L. (2023). Risk stratification of acute pulmonary embolism.. J. Thromb. Haemost..

[4] Kucher N., Rossi E., De Rosa M., Goldhaber S.Z. (2006). Massive pulmonary embolism.. Circulation.

[5] Secemsky E., Chang Y., Jain C.C., Beckman J.A., Giri J., Jaff M.R., Rosenfield K., Rosovsky R., Kabrhel C., Weinberg I. (2018). Contemporary management and outcomes of patients with massive and submassive pulmonary embolism.. Am. J. Med..

[6] Yamamoto T. (2018). Management of patients with high-risk pulmonary embolism: A narrative review.. J. Intensive Care.

[7] Saric M., Armour A.C., Arnaout M.S., Chaudhry F.A., Grimm R.A., Kronzon I., Landeck B.F., Maganti K., Michelena H.I., Tolstrup K. (2016). Guidelines for the use of echocardiography in the evaluation of a cardiac source of embolism.. J. Am. Soc. Echocardiogr..

[8] Goldhaber S.Z., Elliott C.G. (2003). Acute pulmonary embolism: Part i: Epidemiology, pathophysiology, and diagnosis.. Circulation.

[9] Santolicandro A., Prediletto R., Fornai E., Formichi B., Begliomini E., Giannella-Neto A., Giuntini C. (1995). Mechanisms of hypoxemia and hypocapnia in pulmonary embolism.. Am. J. Respir. Crit. Care Med..

[10] Kaplovitch E., Shaw J.R., Douketis J. (2020). Thrombolysis in pulmonary embolism.. Crit. Care Clin..

[11] Ortel T.L., Neumann I., Ageno W., Beyth R., Clark N.P., Cuker A., Hutten B.A., Jaff M.R., Manja V., Schulman S., Thurston C., Vedantham S., Verhamme P., Witt D.M., D Florez I., Izcovich A., Nieuwlaat R., Ross S., J Schünemann H., Wiercioch W., Zhang Y., Zhang Y. (2020). American society of hematology 2020 guidelines for management of venous thromboembolism: Treatment of deep vein thrombosis and pulmonary embolism.. Blood Adv..

[12] Guyatt G.H., Akl E.A., Crowther M., Gutterman D.D., Schuünemann H.J. (2012). American college of chest physicians antithrombotic therapy and prevention of thrombosis panel. Executive summary.. Chest.

[13] Dudzinski D.M., Giri J., Rosenfield K. (2017). Interventional treatment of pulmonary embolism.. Circ. Cardiovasc. Interv..

[14] Goldhaber S.Z., Come P.C., Lee R.T., Braunwald E., Parker J.A., Haire W.D., Feldstein M.L., Miller M., Toltzis R., Smith J.L., Taveira de Silva A.M., Mogtader A., McDonough T.J. (1993). Alteplase versus heparin in acute pulmonary embolism: Randomised trial assessing right-ventricular function and pulmonary perfusion.. Lancet.

[15] Dalla-Volta S., Palla A., Santolicandro A., Giuntini C., Pengo V., Visioli O., Zonzin P., Zanuttini D., Barbaresi F., Agnelli G., Morpurgo M., Marini M.G., Visani L. (1992). Paims 2: Alteplase combined with heparin versus heparin in the treatment of acute pulmonary embolism. plasminogen activator italian multicenter study 2.. J. Am. Coll. Cardiol..

[16] Marti C., John G., Konstantinides S., Combescure C., Sanchez O., Lankeit M., Meyer G., Perrier A. (2015). Systemic thrombolytic therapy for acute pulmonary embolism: A systematic review and meta-analysis.. Eur. Heart J..

[17] Götzinger F., Lauder L., Sharp A.S.P., Lang I.M., Rosenkranz S., Konstantinides S., Edelman E.R., Böhm M., Jaber W., Mahfoud F. (2023). Interventional therapies for pulmonary embolism.. Nat. Rev. Cardiol..

[18] Kuo W.T., Banerjee A., Kim P.S., DeMarco F.J., Levy J.R., Facchini F.R., Unver K., Bertini M.J., Sista A.K., Hall M.J., Rosenberg J.K., De Gregorio M.A. (2015). Pulmonary embolism response to fragmentation, embolectomy, and catheter thrombolysis (perfect).. Chest.

[19] Tapson V.F., Sterling K., Jones N., Elder M., Tripathy U., Brower J., Maholic R.L., Ross C.B., Natarajan K., Fong P., Greenspon L., Tamaddon H., Piracha A.R., Engelhardt T., Katopodis J., Marques V., Sharp A.S.P., Piazza G., Goldhaber S.Z. (2018). A randomized trial of the optimum duration of acoustic pulse thrombolysis procedure in acute intermediate-risk pulmonary embolism.. JACC Cardiovasc. Interv..

[20] Piazza G., Hohlfelder B., Jaff M.R., Ouriel K., Engelhardt T.C., Sterling K.M., Jones N.J., Gurley J.C., Bhatheja R., Kennedy R.J., Goswami N., Natarajan K., Rundback J., Sadiq I.R., Liu S.K., Bhalla N., Raja M.L., Weinstock B.S., Cynamon J., Elmasri F.F., Garcia M.J., Kumar M., Ayerdi J., Soukas P., Kuo W., Liu P.Y., Goldhaber S.Z. (2015). A prospective, single-arm, multicenter trial of ultrasound-facilitated, catheter-directed, low-dose fibrinolysis for acute massive and submassive pulmonary embolism.. JACC Cardiovasc. Interv..

[21] Fukuda I., Daitoku K. (2017). Surgical embolectomy for acute pulmonary thromboembolism.. Ann. Vasc. Dis..

[22] Goldberg J.B., Giri J., Kobayashi T., Ruel M., Mittnacht A.J.C., Rivera-Lebron B., DeAnda A., Moriarty J.M., MacGillivray T.E. (2023). Surgical management and mechanical circulatory support in high-risk pulmonary embolisms: Historical context, current status, and future directions: A scientific statement from the american heart association.. Circulation.

[23] Keeling W.B., Sundt T., Leacche M., Okita Y., Binongo J., Lasajanak Y., Aklog L., Lattouf O.M. (2016). Outcomes after surgical pulmonary embolectomy for acute pulmonary embolus: A multi-institutional study.. Ann. Thorac. Surg..

[24] Lee T., Itagaki S., Chiang Y.P., Egorova N.N., Adams D.H., Chikwe J. (2018). Survival and recurrence after acute pulmonary embolism treated with pulmonary embolectomy or thrombolysis in new york state, 1999 to 2013.. J. Thorac. Cardiovasc. Surg..

[25] Yuriditsky E., Bakker J., Alviar C.L., Bangalore S., Horowitz J.M. (2024). Venoarterial extracorporeal membrane oxygenation in high-risk pulmonary embolism: A narrative review.. J. Crit. Care.

[26] Cooley D.A., Beall A.C., Alexander J.K. (1961). Acute massive pulmonary embolism. successful surgical treatment using temporary cardiopulmonary bypass.. JAMA.

[27] Pasrija C., Kronfli A., George P., Raithel M., Boulos F., Herr D.L., Gammie J.S., Pham S.M., Griffith B.P., Kon Z.N. (2018). Utilization of veno-arterial extracorporeal membrane oxygenation for massive pulmonary embolism.. Ann. Thorac. Surg..

[28] Baldetti L., Beneduce A., Cianfanelli L., Falasconi G., Pannone L., Moroni F., Venuti A., Sacchi S., Gramegna M., Pazzanese V., Calvo F., Gallone G., Pagnesi M., Cappelletti A.M. (2021). Use of extracorporeal membrane oxygenation in high‐risk acute pulmonary embolism: A systematic review and meta‐analysis.. Artif. Organs.

[29] Meneveau N., Guillon B., Planquette B., Piton G., Kimmoun A., Gaide-Chevronnay L., Aissaoui N., Neuschwander A., Zogheib E., Dupont H., Pili-Floury S., Ecarnot F., Schiele F., Deye N., de Prost N., Favory R., Girard P., Cristinar M., Ferré A., Meyer G., Capellier G., Sanchez O. (2018). Outcomes after extracorporeal membrane oxygenation for the treatment of high-risk pulmonary embolism: A multicentre series of 52 cases.. Eur. Heart J..

[30] Farmakis I.T., Sagoschen I., Barco S., Keller K., Valerio L., Wild J., Giannakoulas G., Piazza G., Konstantinides S.V., Hobohm L. (2024). Extracorporeal membrane oxygenation and reperfusion strategies in high-risk pulmonary embolism hospitalizations.. Crit. Care Med..

[31] Corsi F., Lebreton G., Bréchot N., Hekimian G., Nieszkowska A., Trouillet J.L., Luyt C.E., Leprince P., Chastre J., Combes A., Schmidt M. (2017). Life-threatening massive pulmonary embolism rescued by venoarterial-extracorporeal membrane oxygenation.. Crit. Care.

[32] Konstantinides S.V., Meyer G., Becattini C., Bueno H., Geersing G.J., Harjola V.P., Huisman M.V., Humbert M., Jennings C.S., Jiménez D., Kucher N., Lang I.M., Lankeit M., Lorusso R., Mazzolai L., Meneveau N., Ní Áinle F., Prandoni P., Pruszczyk P., Righini M., Torbicki A., Van Belle E., Zamorano J.L., Galié N., Gibbs J.S.R., Aboyans V., Ageno W., Agewall S., Almeida A.G., Andreotti F., Barbato E., Bauersachs J., Baumbach A., Beygui F., Carlsen J., De Carlo M., Delcroix M., Delgado V., Subias P.E., Fitzsimons D., Gaine S., Goldhaber S.Z., Gopalan D., Habib G., Halvorsen S., Jenkins D., Katus H.A., Kjellström B., Lainscak M., Lancellotti P., Lee G., Le Gal G., Messas E., Morais J., Petersen S.E., Petronio A.S., Piepoli M.F., Price S., Roffi M., Salvi A., Sanchez O., Shlyakhto E., Simpson I.A., Stortecky S., Thielmann M., Noordegraaf A.V., Becattini C., Bueno H., Geersing G-J., Harjola V-P., Huisman M.V., Humbert M., Jennings C.S., Jiménez D., Kucher N., Lang I.M., Lankeit M., Lorusso R., Mazzolai L., Meneveau N., Ní Áinle F., Prandoni P., Pruszczyk P., Righini M., Torbicki A., VanBelle E., LuisZamorano J., Windecker S., Aboyans V., Baigent C., Collet J-P., Dean V., Delgado V., Fitzsimons D., Gale C.P., Grobbee D., Halvorsen S., Hindricks G., Iung B., Jüni P., Katus H.A., Landmesser U., Leclercq C., Lettino M., Lewis B.S., Merkely B., Mueller C., Petersen S.E., Sonia Petronio A., Richter D.J., Roffi M., Shlyakhto E., Simpson I.A., Sousa-Uva M., Touyz R.M., Hammoudi N., Hayrapetyan H., Mascherbauer J., Ibrahimov F., Polonetsky O., Lancellotti P., Tokmakova M., Skoric B., Michaloliakos I., Hutyra M., Mellemkjaer S., Mostafa M., Reinmets J., Jääskeläinen P., Angoulvant D., Bauersachs J., Giannakoulas G., Zima E., Vizza C.D., Sugraliyev A., Bytyçi I., Maca A., Ereminiene E., Huijnen S., Xuereb R., Diaconu N., Bulatovic N., Asfalou I., Bosevski M., Halvorsen S., Sobkowicz B., Ferreira D., Petris A.O., Moiseeva O., Zavatta M., Obradovic S., Šimkova I., Radsel P., Ibanez B., Wikström G., Aujesky D., Kaymaz C., Parkhomenko A., Pepke-Zaba J. (2020). 2019 esc guidelines for the diagnosis and management of acute pulmonary embolism developed in collaboration with the European Respiratory Society (ERS).. Eur. Heart J..

[33] Panchal A.R., Bartos J.A., Cabañas J.G., Donnino M.W., Drennan I.R., Hirsch K.G., Kudenchuk P.J., Kurz M.C., Lavonas E.J., Morley P.T., O’Neil B.J., Peberdy M.A., Rittenberger J.C., Rodriguez A.J., Sawyer K.N., Berg K.M., Arafeh J., Benoit J.L., Chase M., Fernandez A., de Paiva E.F., Fischberg B.L., Flores G.E., Fromm P., Gazmuri R., Gibson B.C., Hoadley T., Hsu C.H., Issa M., Kessler A., Link M.S., Magid D.J., Marrill K., Nicholson T., Ornato J.P., Pacheco G., Parr M., Pawar R., Jaxton J., Perman S.M., Pribble J., Robinett D., Rolston D., Sasson C., Satyapriya S.V., Sharkey T., Soar J., Torman D., Von Schweinitz B., Uzendu A., Zelop C.M., Magid D.J. (2020). Part 3: Adult basic and advanced life support: 2020 American Heart Association Guidelines for cardiopulmonary resuscitation and emergency cardiovascular care.. Circulation.

[34] Seaton A., Hodgson L.E., Creagh-Brown B., Pakavakis A., Wyncoll D.L.A., Doyle JF J.F. (2017). The use of veno-venous extracorporeal membrane oxygenation following thrombolysis for massive pulmonary embolism.. J. Intensive Care Soc..

[35] Kmiec L., Philipp A., Floerchinger B., Lubnow M., Unterbuchner C., Creutzenberg M., Lunz D., Mueller T., Schmid C., Camboni D. (2020). Extracorporeal membrane oxygenation for massive pulmonary embolism as bridge to therapy.. ASAIO J..

[36] Rosovsky R., Zhao K., Sista A., Rivera-Lebron B., Kabrhel C. (2019). Pulmonary embolism response teams: Purpose, evidence for efficacy, and future research directions.. Res. Pract. Thromb. Haemost..

[37] Dudzinski D.M., Piazza G. (2016). Multidisciplinary pulmonary embolism response teams.. Circulation.

[38] Kabrhel C., Rosovsky R., Channick R., Jaff M.R., Weinberg I., Sundt T., Dudzinski D.M., Rodriguez-Lopez J., Parry B.A., Harshbarger S., Chang Y., Rosenfield K. (2016). A multidisciplinary pulmonary embolism response team.. Chest.

[39] Martin K.A., Molsberry R., Cuttica M.J., Desai K.R., Schimmel D.R., Khan S.S. (2020). Time trends in pulmonary embolism mortality rates in the united states, 1999 to 2018.. J. Am. Heart Assoc..

[40] Elbadawi A., Mentias A., Elgendy I.Y., Mohamed A.H., Syed M.H.Z., Ogunbayo G.O., Olorunfemi O., Gosev I., Prasad S., Cameron S.J. (2019). National trends and outcomes for extra-corporeal membrane oxygenation use in high-risk pulmonary embolism.. Vasc. Med..

[41] Bhalla A., Attaran R. (2020). Mechanical circulatory support to treat pulmonary embolism: Venoarterial extracorporeal membrane oxygenation and right ventricular assist devices.. Tex. Heart Inst. J..

[42] Kaso E.R., Pan J.A., Salerno M., Kadl A., Aldridge C., Haskal Z.J., Kennedy J.L.W., Mazimba S., Mihalek A.D., Teman N.R., Giri J., Aronow H.D., Sharma A.M. (2022). Venoarterial extracorporeal membrane oxygenation for acute massive pulmonary embolism: A meta-analysis and call to action.. J. Cardiovasc. Transl. Res..

[43] Tsai H.Y., Wang Y.T., Lee W.C., Yen H.T., Lo C.M., Wu C.C., Huang K.R., Chen Y.C., Sheu J.J., Chen Y.Y. (2022). Efficacy and safety of veno-arterial extracorporeal membrane oxygenation in the treatment of high-risk pulmonary embolism: A retrospective cohort study.. Front. Cardiovasc. Med..

[44] Karami M., Mandigers L., Miranda D.D.R., Rietdijk W.J.R., Binnekade J.M., Knijn D.C.M., Lagrand W.K., den Uil C.A., Henriques J.P.S., Vlaar A.P.J. (2021). Survival of patients with acute pulmonary embolism treated with venoarterial extracorporeal membrane oxygenation: A systematic review and meta-analysis.. J. Crit. Care.

[45] Scott J.H., Gordon M., Vender R., Pettigrew S., Desai P., Marchetti N., Mamary A.J., Panaro J., Cohen G., Bashir R., Lakhter V., Roth S., Zhao H., Toyoda Y., Criner G., Moores L., Rali P. (2021). Venoarterial extracorporeal membrane oxygenation in massive pulmonary embolism-related cardiac arrest: A systematic review.. Crit. Care Med..

[46] Hobohm L., Sagoschen I., Habertheuer A., Barco S., Valerio L., Wild J., Schmidt F.P., Gori T., Münzel T., Konstantinides S., Keller K. (2022). Clinical use and outcome of extracorporeal membrane oxygenation in patients with pulmonary embolism.. Resuscitation.

[47] Oh Y.N., Oh D.K., Koh Y., Lim C.M., Huh J.W., Lee J.S., Jung S.H., Kang P.J., Hong S.B. (2019). Use of extracorporeal membrane oxygenation in patients with acute high-risk pulmonary embolism: A case series with literature review.. Acute Crit. Care.

[48] Guliani S., Das Gupta J., Osofsky R., Kraai E.P., Mitchell J.A., Dettmer T.S., Wray T.C., Tawil I., Rana M.A., Marinaro J. (2021). Venoarterial extracorporeal membrane oxygenation is an effective management strategy for massive pulmonary embolism patients.. J. Vasc. Surg. Venous Lymphat. Disord..

[49] Giraud R., Laurencet M., Assouline B., De Charrière A., Banfi C., Bendjelid K. (2021). Can va-ecmo be used as an adequate treatment in massive pulmonary embolism?. J. Clin. Med..

[50] Prasad N.K., Boyajian G., Tran D., Shah A., Jones K.M., Madathil R.J., Deatrick K.B., Cires-Drouet R., Kaczorowski D.J. (2022). Veno-arterial extracorporeal membrane oxygenation for pulmonary embolism after systemic thrombolysis.. Semin. Thorac. Cardiovasc. Surg..

[51] Al-Bawardy R., Rosenfield K., Borges J., Young M.N., Albaghdadi M., Rosovsky R., Kabrhel C. (2019). Extracorporeal membrane oxygenation in acute massive pulmonary embolism: A case series and review of the literature.. Perfusion.

[52] George B., Parazino M., Omar H.R., Davis G., Guglin M., Gurley J., Smyth S. (2018). A retrospective comparison of survivors and non-survivors of massive pulmonary embolism receiving veno-arterial extracorporeal membrane oxygenation support.. Resuscitation.

[53] Assouline B., Assouline-Reinmann M., Giraud R., Levy D., Saura O., Bendjelid K., Combes A., Schmidt M. (2022). Management of high-risk pulmonary embolism: What is the place of extracorporeal membrane oxygenation?. J. Clin. Med..

[54] Ius F., Hoeper M.M., Fegbeutel C., Kühn C., Olsson K., Koigeldiyev N., Tudorache I., Warnecke G., Optenhöfel J., Puntigam J.O., Schäfer A., Meyer B.C., Hinrichs J.B., Bauersachs J., Haverich A., Cebotari S. (2019). Extracorporeal membrane oxygenation and surgical embolectomy for high-risk pulmonary embolism.. Eur. Respir. J..

